# Behavioural and chemical evidence for multiple colonisation of the Argentine ant, *Linepithema humile*, in the Western Cape, South Africa

**DOI:** 10.1186/1472-6785-11-6

**Published:** 2011-02-03

**Authors:** Natasha P Mothapo, Theresa C Wossler

**Affiliations:** 1 DST/NRF Centre of Excellence for Invasion Biology, Department of Botany and Zoology, University of Stellenbosch, Private Bag X1, Matieland, 7602, South Africa

## Abstract

**Background:**

The Argentine ant, *Linepithema humile*, is a widespread invasive ant species that has successfully established in nearly all continents across the globe. Argentine ants are characterised by a social structure known as unicoloniality, where territorial boundaries between nests are absent and intraspecific aggression is rare. This is particularly pronounced in introduced populations and results in the formation of large and spatially expansive supercolonies. Although it is amongst the most well studied of invasive ants, very little work has been done on this ant in South Africa. In this first study, we investigate the population structure of Argentine ants in South Africa. We use behavioural (aggression tests) and chemical (CHC) approaches to investigate the population structure of Argentine ants within the Western Cape, identify the number of supercolonies and infer number of introductions.

**Results:**

Both the aggression assays and chemical data revealed that the Western Cape Argentine ant population can be divided into two behaviourally and chemically distinct supercolonies. Intraspecific aggression was evident between the two supercolonies of Argentine ants with ants able to discriminate among conspecific non-nestmates. This discrimination is linked to the divergence in cuticular hydrocarbon profiles of ants originating from the two supercolonies.

**Conclusions:**

The presence of these two distinct supercolonies is suggestive of at least two independent introductions of this ant within the Western Cape. Moreover, the pattern of colonisation observed in this study, with the two colonies interspersed, is in agreement with global patterns of Argentine ant invasions. Our findings are of interest because recent studies show that Argentine ants from South Africa are different from those identified in other introduced ranges and therefore provide an opportunity to further understand factors that determine the distributional and spread patterns of Argentine ant supercolonies.

## Background

Many species have been accidentally introduced into areas outside their geographic distribution as a direct consequence of human trade [1]. Indeed, the influx of exotic species has significantly increased globally with increased trade, highlighting the important role of human activity as a major driver of biological invasions [2-4]. Once established, invasive species have wide ranging impacts on the ecosystem including the displacement of native biota, disruption of ecosystem function in natural systems [5-7] as well as severe economic impacts in agriculture and forestry sectors [8]. Thus, invasive species are considered amongst the most significant threats to biodiversity globally [9].

Ants are regarded among the most damaging of invasive species largely due to the important roles they play in ecosystems globally [10-14]. Indeed five ant species are listed amongst the top 100 worst invaders in the world [12] and many have already established in most continents on the globe [15,16]. Although many of these ants show strong affinity for human modified habitats where there is high resource availability and limited biotic resistance [17], several have successfully penetrated into natural communities [10,16,18,19].

The Argentine ant, *Linepitheman humile*, originates from South America and has successfully established on six continents and several oceanic islands. Their colony structure is the best studied of invasive ant species [20-24]. Similar to other invasive ants, Argentine ants are characterised by a social structure known as unicoloniality, where territorial boundaries are absent and intraspecific aggression between physically separate nests is rare [25-29]. Introduced populations form supercolonies that extend over thousands of kilometres [20,22,24,30-32] compared to their native range [33,34]. Ants from spatially separate nests within a supercolony treat each other as nestmates; however, ants from different supercolonies show pronounced aggression [20,30,35-37]. It is well established that aggression between ants from different colonies is determined by genetic similarity and similarity in cuticular hydrocarbon profiles (CHC) [23,29], which represent recognition signals in ants and other insects [30,38-40].

Argentine ants in the introduced ranges are characterised by widespread acceptance of non-nestmate conspecifics, chemical and genetic similarity amongst distant populations [20,23,25,30,31,35,41]. Thus, mutually tolerant supercolonies sharing both chemical (CHC profiles) and genetic similarity are expected to originate from the same source colonies in the native range [34,41]. This knowledge has been widely used to ascertain supercolony identity in both native and introduced ranges of the Argentine ant [22,31,34,35,41,42], and has more recently been used to investigate introduction history of these ants and other invasive ants [22,43,44]. Several studies on the population structure of Argentine ants in most parts of the introduced range revealed the presence of multiple supercolonies, usually a single large supercolony with smaller supercolonies [30,36]. The presence of different supercolonies within a geographic area is thought to indicate multiple introduction events [35,44]. We use a combination of behavioural (aggression bioassays) and chemical (cuticular hydrocarbons) approaches to investigate the population structure of Argentine ants in the Western Cape, South Africa. Supercolony boundaries have been determined at local scales in both native and introduced ranges [20,22,30-34,41], and more recently across continents [24,41,45] however, this is not true for South Africa.

Since introduction into the Western Cape, South Africa in the early 1900s [46], Argentine ants have successfully spread throughout the region in both urban and natural environments [47,48]. The impacts of the invasion on the Fynbos biome of South Africa, a biodiversity hotspot, are similar to other introduced areas [49-51]. Argentine ants displace native ants and other arthropod fauna [52,53], disrupting important plant-ant interactions [54] and leading to the alteration of ecosystem functioning and cascading effects on other trophic levels [55]. Previous studies suggest that the Argentine ant has colonised South Africa multiple times, however, very little work has been done on this ant in South Africa and large scale data is lacking despite its clear importance. Tsutsui *et al.*, (2001) found that Argentine ants from three localities in the Western Cape, South Africa, form two genetically different groups. This suggested that Argentine ants have been multiply introduced into South Africa and that more than one supercolony exists. Recently, Vogel *et al. *(2010) and Van Wilgenburg *et al. *(2010) showed that ants from Stellenbosch do not form part of the global large supercolony as identified in both these studies. Thus, the aims of this study are to [1] determine the population structure of Argentine ants within the Western Cape, South Africa (the point of entry for this ant); [2] to identify the number of supercolonies within this region, infer the number of introduction and relate the findings with what is observed in other introduced ranges.

## Methods

### Collection of Argentine ants

**Figure 1:**
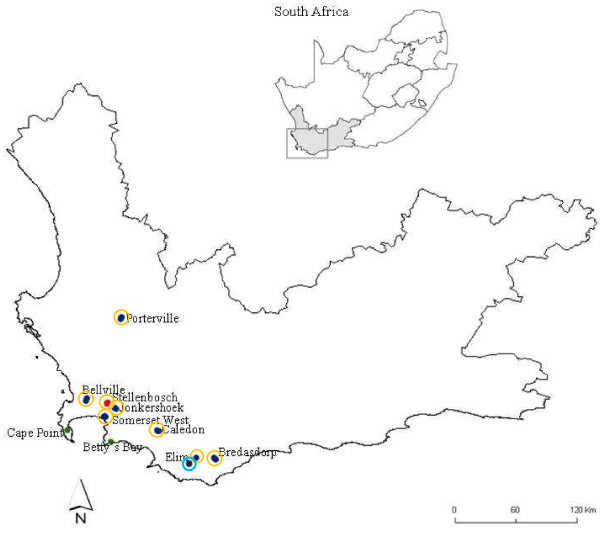
**Map of Western Cape, South Africa and the sites used in this study**. Map of the Western Cape region, South Africa with the eight sites from which Argentine ants were collected for this study, including sites from previous studies ("green circle") Tsutsui *et al.*, 2001 (Cape point, Betty's Bay and Caledon); ("red circle") Vogel *et al.*, 2010 (Stellenbosch) and Van Wilgenburg *et al.*, 2010 (Stellenbosch). Ants were collected from Porterville in the North right down to Elim in the South.

Argentine ants were collected from eight sites in the Western Cape region of South Africa: Stellenbosch, Somerset West, Jonkershoek, Bellville, Caledon, Bredasdorp, Elim and Porterville from September to October 2007 (Table 1, Figure 1). The sites sampled ranged over 900km from the northern to the southern part of the Western Cape. For each site, we collected three nests (consisting of queens, workers and brood) at least 500m apart to avoid collecting ants from the same nests. Nests were collected in plastic containers (30cm × 2cm × 8cm) lined with Fluon (Fluoropolymer Dispersion, Whitford Plastics Ltd, England) one quarter down from the brim to prevent ants escaping. Prior to behavioural assays, ants were provided with 0.25 M sugar water only.

**Table 1:** Collection sites for Argentine ants in the Western Cape, South Africa

Nest	Latitude	Longitude
Stellenbosch 1	33° 55.945'	018° 51.825'
Stellenbosch 2	33° 55.746'	018° 51.889'
Stellenbosch 3	33° 55.959'	018° 51.990'
Bellville 1	33° 55.940'	018° 51.609'
Bellville 2	33° 54.132'	018° 37.593'
Bellville 3	33° 52.346'	018° 38.200'
Porterville 1	33° 00.683'	019° 00.511'
Porterville 2	32° 59.072'	018° 01.457'
Porterville 3	32° 59.078'	019° 01.392'
Elim 1	34° 35.547'	019° 45.589'
Elim 2	34° 35.277'	019° 45.445'
Elim 3	34° 31.751'	019° 50.112'
Bredasdorp 1	34° 32.258'	020° 02.760'
Bredasdorp 2	34° 32.607'	020° 02.892'
Bredasdorp 3	34° 31.830'	020° 02.106'
Caledon 1	34° 14.355'	019° 25.699'
Caledon 2	34° 13.616'	019° 24.561'
Caledon 3	34° 14.045'	019° 25.175'
Somerset west 1	34° 04.173'	018° 49.810'
Somerset west 2	34° 05.045'	018° 49.326'
Somerset west 3	34° 04.669'	018° 50.717'
Jonkershoek 1	33° 58.819'	018° 56.738'
Jonkershoek 2	33° 59.482'	018° 57.251'
Jonkershoek 3	33° 58.733'	018° 56.982'
		

List and GPS coordinates of field sites where Argentine ants were collected.

### Behavioural assays

Individual ants were paired from the same nest (controls), between nests within the same site as well as between nests from different sites. Pairwise aggression tests, adapted from earlier studies [20,25,30] were used to assess the pattern of intraspecific aggression among Argentine ants within the Western Cape. Single, randomly picked workers from each of the two nests were paired in an 8ml glass pill vial lined with Fluon (one quarter down from the brim). Behavioural interactions between the two ants were observed and recorded over 10 minutes and scored according to escalating aggression on a scale from 0 - 4. Behavioural interactions were categorised as follows: 0 - ignore, 1- antennate with no aggressive response, 2 - retract or avoidance, 3 - aggression (such as biting, lunging, pulling and mandible gaping) and 4 - prolonged aggression or fighting. Categories 0 to 2 were regarded as non-aggressive while 3 and 4 were aggressive [25]. For each nest pair, the behavioural assay was repeated ten times while each worker was used only once. The nest origin was unknown to the observer.

### Chemical analyses

Ten workers from the same nest were washed in 100 μl of hexane for 10 minutes, followed by a second two minute rinse in another 100 μl of hexane. The two samples were combined to produce a 200 μl cuticular lipid extract. To purify the extract, silica gel minicolumns were constructed from glass Pasteur pipette tubes filled with ± 500 mg of silica gel (grade 13,30-200 mesh, SIGMA-Aldrich, USA), plugged with glass wool and pre-wetted with 2ml hexane prior to loading the cuticular lipid extract (here forth, CHC extract) [56-59]. The CHC extract was concentrated under a stream of nitrogen to 100 μl and then loaded onto the pre-wetted silica gel minicolumn. The hydrocarbon fraction was eluted with 3ml of hexane, evaporated to dryness under a stream of nitrogen and redissolved in 25 μl hexane. A volume of 1 μl of the extract was injected in a Gas Chromatograph (Agilent3850) fitted with a splitless inlet, flame-ionisation detection and a DB-5 capillary column (30m × 0.32mm × 0.25 μm film thickness, Agilent Technologies, CA). The injection port and the detector were set at 290°C and 320°C, respectively. Helium was used as the carrier gas at 30.4 ml/min and nitrogen as the make-up gas. The following temperature program was used: oven temperature was held at 80°C for 2 minutes, and then increased to 270°C at a rate of 10°C/min then raised to 310°C at 3°C/min and finally held at 310°C for 20 minutes. Electron impact mass spectra (Agilent 5975B mass spectrometer) were used as the means of identifying the peaks (compounds) on the chromatograms. The compounds were identified by comparing their mass spectra with those of pure compounds accessed via the WILEY and NIST (National Institute of Standards and Technology) databases. Only compounds with a match of 90% or more to those accessed in the library were positively identified. All compounds in trace quantities were not included because their abundances could not be calculated.

### Statistical analyses

#### Behavioural assays

Behavioural categories were converted to binary data, aggression vs. non-aggression. In many behavioural studies, the scores are averaged to get a single number or aggression index that is thought to represent the level of aggression per nest pair in each trial [25,60]; and used to statistically analyse the data. However, converting categorical data into mean behavioural or aggression indices is thought to conceal some subtle behavioural differences between trials [42]. Therefore, two non-parametric approaches were used to analyse the behavioural data: (i) Chi-square tests were used to compare aggression between nestmates and non-nestmates within sites. (ii) Multivariate data analyses were performed in PRIMER (Plymouth Routines in Multivariate Ecological Research, version 5.2.9, 2004: Plymouth Marine Laboratory, UK) to assess the differences in the level of aggressive interactions for nests between sites. An ordination analysis was conducted using non-metric Multidimensional Scaling (hereafter MDS) plots that score and display categories based on their similarity or dissimilarity [61]. The stress value on the MDS plot is a measure of the goodness of fit and is dependent on the dimensions of the data used. A stress value below 0.7 is an indication of a good fit [62]. Bray-Curtis coefficients were used to calculate the similarity matrix [63] and an Analysis of Similarity (ANOSIM) test, based on 1000 permutations, was used to assess the significance of the separation of aggressive interactions between the groups on the MDS plot. Global R values closer or equal to zero indicate strong similarity between the test groups and those closer to or equal to one indicate very strong differences between the test groups. Statistical significance was accepted at p < 0.05.

#### Chemical analyses

Forty cuticular compounds were separated and identified by Gas Chromatography/Mass Spectrometry (GC/MS), and the peak areas were standardised to 100% by calculating the percentage contribution of each compound to the cuticular hydrocarbon blend. The proportion of the relative compounds was calculated as the ratio of that compound relative to the other 39 compounds. Because peak areas represent compositional data, the standardised peak areas were transformed to logcontrasts using Aitchison's (1986) [64] formula: Z_ij_=ln[Y_ij_/g(Y_j_)], where Z_ij _is the standardised peak area *i*, for individual *j*, Y_ij _is the peak area *i *for individual *j*, and g(Y_j_) is the geometric mean of all peaks for individual *j*. Multivariate data analyses were performed using SPSS 17.0 software. The standardised peak areas were subjected to a Principal Components Analysis (PCA), with varimax rotation, to reduce the number of describing variables. The extracted PCA factors were further subjected to a Discriminant Analysis (DA) to determine whether the behaviourally defined Argentine ant colonies could also be discriminated on the basis of their CHC profiles.

## Results

### Behavioural Assays

**Figure 2:**
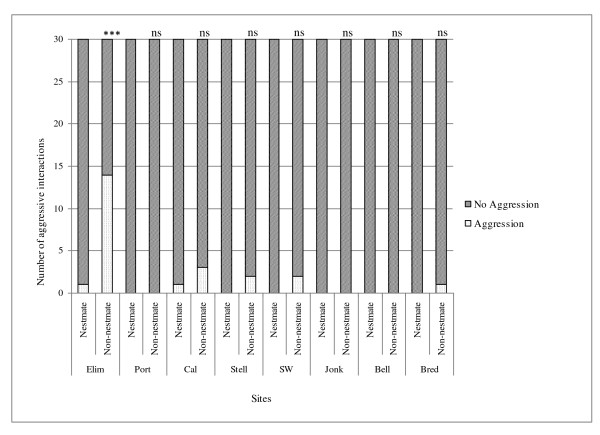
**Behavioural interactions of Argentine ants within sites**. Frequency of aggressive and non-aggressive behavioural interactions between nestmates and non-nestmates within each site. The frequency represents the total number of interactions (aggressive and non-aggressive) of all three nests within a site for within and between nest interactions observed over 10 minutes. Levels of significance shown by (*ns*) p > 0.05 and (***) P < 0.001 (Chi-Square test). Locality abbreviations: Elim = Elim, Port = Porterville, Cal = Caledon, Stell = Stellenbosch, SW = Somerset West, Jonk = Jonkershoek, Bell = Bellville, and Bred = Bredasdorp.

**Figure 3:**
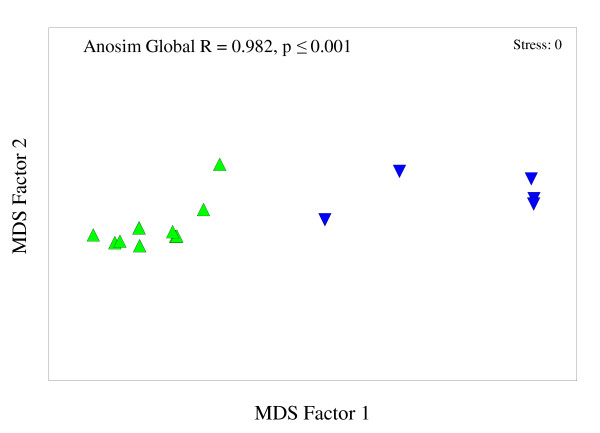
**Behavioural interactions of Argentine ants from different sites**. Non-metric Multidimensional Scaling (MDS) plot showing all aggressive and non-aggressive behavioural interactions for paired nests between sites. Analyses were conducted using the categories aggression and non-aggression. The separation incorporated colony interactions that included *Elim 3 *and those that did not. All interactions that included *Elim 3 *("green triangle") and those that excluded *Elim 3 *("blue trianlgle") grouped separately indicating a distinct boundary between Elim and all other sites.

Aggression tests revealed that Argentine ants within the Western Cape are unicolonial, as ants from distant sites were mutually tolerant. Intraspecific aggression was rare between ants from the same site (Figure 2). One nest in Elim, *Elim 3*, was aggressive to all other nests within this site (*Elim 1 *and *Elim 2*; Figure 2).Consequently; two nests (*Elim 2 *- non-aggressive; *Elim 3 *- aggressive) were selected and treated independently in further analyses. There was very little aggression observed between ants from different sites despite large geographic distances separating them (Table 2). Only interactions that included ants from Elim resulted in significantly high levels of injurious aggression (Table 2). The MDS revealed two groups, those interactions including ants from *Elim 3 *and those that did not (Global R = 0.982, p < 0.001; Figure 3), suggesting that a behavioural boundary exists between ants from *Elim 3 *and all the other sites included in this study. In assays between non-aggressive non-nestmates, ants spent most of their time antennating or self-grooming. Furthermore, these data show no relationship between aggression and distance between nests, since ants remained non-aggressive despite large geographical distances separating them (see Figure 1).

**Table 2:** Aggressive interactions between ants from different sites

	SW	Cal	Stel	EL3	EL2	Port	Jonk	Bell	Bred
**SW**									
**Cal**	1/10^ns^								
**Stel**	0/10^ns^	0/10^ns^							
**El3**	7/10***	7/10***	8/10***						
**EL2**	3/10*	4/10*	0/10^ns^	9/10***					
**Port**	0/10^ns^	0/10^ns^	0/10^ns^	8/10***	1/10^ns^				
**Jonk**	1/10^ns^	0/10^ns^	0/10^ns^	9/10***	4/10*	0/10^ns^			
**Bell**	1/10^ns^	0/10^ns^	0/10^ns^	6/10**	0/10^ns^	0/10^ns^	0/10^ns^		
**Bred**	1/10^ns^	2/10^ns^	0/10^ns^	8/10***	3/10*	0/10^ns^	0/10^ns^	0/10^ns^	

Frequency of aggression between ants from different sites. Levels of significance based on Chi-Square test shown by (ns) P > 0.05, (*) P < 0.05, (**) P < 0.01 and (***) P <0.001. Degrees of Freedom (*df *= 1). Site abbreviations: SW = Somerset West, Cal = Caledon, Stel = Stellenbosch, EL3 = Elim 3, EL2 = Elim 2, Jonk = Jonkershoek, Bell = Bellville and Bred = Bredasdorp.

### Chemical analyses

**Figure 4:**
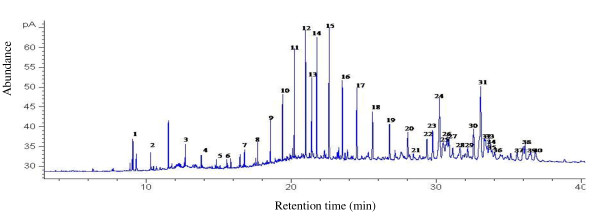
**Chromatogram of Argentine ants from Western Cape, South Africa**. A representative gas chromatogram of a cuticular hydrocarbon profile of field collected Argentine ants in the Western Cape. Chromatogram of Elim nest 2, See Table 1 for peak identification.

Forty compounds were separated and thirty eight of them identified in the CHC profile of field populations of Argentine ant (see Table 3 for compound identification). The profiles are characterised by a series of linear alkanes (retention times 9-28 mins) followed by clusters of long-chained and methyl-branched hydrocarbons (retention times 30-38 mins) (Figure 4) with chain-lengths ranging from C_13_-C_44 _(Table 3). Saturated long-chained hydrocarbons, namely, hexacosane, heptacosane and octacosane were the most abundant compounds in the profiles of ants from *Stellenbosch*, while 13-methylhentriacontane was abundant in the profiles of ants from *Porterville *and *Bredasdorp*. The cuticular profiles of ants from *Elim 3 *are distinguishable from all other sites in that *Elim 3 *ants have high abundances of the two unknown compounds (peaks 35 and 40) and low abundances of tetratriacontane (peak 24) and heptatriacontane (peak 28) in contrast to other samples (Table 3).

**Table 3:** List of Compounds found in CHC profiles of Argentine ants

Peak	Compound	Elim _(1 + 2)_	Elim 3	Bell	Stell	Cal	Port	SW	Bredas	Jonk
1	Tridecane	1.83 ± 0.45	2.07 ± 0.60	1.73 ± 0.48	1.33 ± 0.75	2.50 ± 0.96	2.19 ± 0.54	2.14 ± 0.63	3.06 ± 0.59	2.75 ± 0.41
2	Tetradecane	0.33 ± 0.22	0.44 ± 0.11	0.34 ± 0.19	0.00	0.06 ± 0.07	0.56 ± 0.28	0.66 ± 0.27	0.58 ± 0.21	0.46 ± 0.21
3	Hexadecane	0.80 ± 0.18	0.74 ± 0.17	0.28 ± 0.11	0.35 ± 0.25	0.87 ± 0.42	0.39 ± 0.27	0.73 ± 0.19	0.38 ± 0.20	0.79 ± 0.22
4	Heptadecane	0.29 ± 0.13	0.47 ± 0.10	0.36 ± 0.10	0.00	0.18 ± 0.13	0.00	0.61 ± 0.19	0.70 ± 0.26	0.93 ± 0.33
5	Octadecane	0.03 ± 0.03	0.17 ± 0.08	0.10 ± 0.06	0.00	0.27 ± 0.26	0.26 ± 0.46	0.22 ± 0.13	0.18 ± 0.11	0.00
6	Nonadecane	0.03 ± 0.04	0.24 ± 0.08	0.13 ± 0.07	0.00	0.24 ± 0.22	0.25 ± 0.35	0.18 ± 0.11	0.22 ± 0.12	0.00
7	Eicosane	0.49 ± 0.13	0.63 ± 0.04	0.52 ± 0.12	0.18 ± 0.17	0.92 ± 0.38	0.66 ± 0.73	0.94 ± 0.36	0.62 ± 0.26	0.50 ± 0.40
8	Heneicosane	0.79 ± 0.17	0.87 ± 0.05	0.94 ± 0.07	0.67 ± 0.70	1.08 ± 0.62	0.79 ± 0.27	2.05 ± 0.63	1.02 ± 0.42	0.64 ± 0.23
9	Docosane	1.82 ± 0.20	1.52 ± 0.11	1.61 ± 0.12	2.04 ± 0.71	4.07 ± 1.66	2.00 ± 0.29	5.88 ± 1.87	2.53 ± 1.18	1.57 ± 0.35
10	Tricosane	2.83 ± 0.25	2.43 ± 0.19	2.71 ± 0.21	4.27 ± 1.10	5.67 ± 1.95	3.15 ± 0.47	7.96 ± 2.29	3.87 ± 1.32	2.89 ± 0.38
11	Tetracosane	4.11 ± 0.29	3.56 ± 0.33	3.80 ± 0.30	6.25 ± 1.29	7.78 ± 2.28	4.88 ± 0.63	10.15 ± 2.62	5.65 ± 1.45	4.23 ± 0.52
12	Pentacosane	5.88 ± 0.49	4.66 ± 0.35	5.25 ± 0.43	6.51 ± 2.55	6.12 ± 1.45	6.41 ± 0.81	5.14 ± 1.70	5.83 ± 1.04	5.63 ± 0.66
13	1,2,benzenedicarboxylic acid	4.34 ± 1.31	2.08 ± 0.30	1.89 ± 0.44	1.53 ± 1.01	1.50 ± 0.69	2.01 ± 0.67	1.53 ± 0.53	3.10 ± 1.03	2.01 ± 0.45
14	Hexacosane	5.51 ± 0.43	4.83 ± 0.37	5.17 ± 0.34	9.53 ± 2.25	7.19 ± 1.46	6.16 ± 0.73	8.33 ± 1.01	6.44 ± 0.80	5.21 ± 0.62
15	Heptacosane	6.30 ± 0.17	6.94 ± 0.42	5.75 ± 0.41	10.88 ± 2.73	7.21 ± 0.48	5.71 ± 0.63	6.32 ± 0.32	5.96 ± 0.88	5.48 ± 0.50
16	Octacosane	4.20 ± 0.19	4.18 ± 0.25	4.80 ± 0.34	7.38 ± 1.79	5.22 ± 0.54	4.92 ± 0.79	4.88 ± 0.26	4.76 ± 0.52	4.45 ± 0.42
17	Nonacosane	4.43 ± 0.24	4.50 ± 0.26	4.99 ± 0.38	6.82 ± 1.31	4.82 ± 0.45	4.63 ± 0.44	4.27 ± 0.40	4.98 ± 0.62	4.46 ± 0.40
18	Triacontane	3.36 ± 0.24	3.70 ± 0.15	4.41 ± 0.60	4.11 ± 1.01	3.75 ± 0.45	3.53 ± 0.38	3.05 ± 0.26	3.50 ± 0.45	3.48 ± 0.49
19	Hentriacontane	3.04 ± 0.23	3.07 ± 0.22	3.73 ± 0.44	2.97 ± 0.65	3.15 ± 0.42	2.81 ± 0.33	2.67 ± 0.34	2.82 ± 0.41	3.13 ± 0.31
20	Dotriacontane	2.72 ± 0.23	3.37 ± 0.48	3.60 ± 0.58	2.33 ± 0.65	2.77 ± 0.42	2.45 ± 0.43	2.23 ± 0.28	2.04 ± 0.47	2.90 ± 0.25
21	13-methylhentriacontane	0.03 ± 0.04	0.26 ± 0.18	0.51 ± 0.41	3.17 ± 3.45	0.44 ± 0.36	13.40 ± 6.91	1.18 ± 0.94	12.97 ± 4.49	3.38 ± 2.64
22	Tritriacontane	2.04 ± 0.19	2.36 ± 0.16	2.45 ± 0.34	1.28 ± 0.49	2.05 ± 0.34	1.68 ± 0.28	1.88 ± 0.30	1.67 ± 0.38	2.11 ± 0.19
23	2,6,10,15 tetramethyl heptadecane	2.75 ± 0.21	1.41 ± 0.18	2.78 ± 0.28	1.56 ± 0.58	1.82 ± 0.60	2.08 ± 0.34	1.89 ± 0.46	2.17 ± 0.46	1.88 ± 0.34
24	Tetratriacontane	8.25 ± 0.80	0.33 ± 0.11	6.38 ± 0.58	7.06 ± 2.44	5.26 ± 1.41	7.71 ± 1.59	4.04 ± 1.29	4.47 ± 1.11	7.68 ± 0.99
25	Pentatriacontane	1.64 ± 0.29	2.91 ± 0.26	2.95 ± 0.46	2.32 ± 1.50	2.63 ± 0.77	2.76 ± 2.09	1.35 ± 0.42	1.27 ± 0.58	2.32 ± 0.82
26	Hexatriacontane	2.81 ± 0.52	1.33 ± 1.33	4.09 ± 0.49	3.03 ± 1.02	2.72 ± 0.76	2.77 ± 0.52	2.74 ± 0.50	2.34 ± 0.45	3.28 ± 0.38
27	13,17,21 trimethylpentatriacontane	2.08 ± 0.36	1.62 ± 1.62	1.15 ± 0.28	0.40 ± 0.35	1.13 ± 0.46	1.19 ± 0.38	0.63 ± 0.35	1.36 ± 0.47	2.35 ± 0.47
28	Heptatriacontane	1.81 ± 0.21	0.10 ± 0.05	1.32 ± 0.21	0.29 ± 0.23	0.78 ± 0.35	0.70 ± 0.28	0.83 ± 0.35	0.63 ± 0.23	1.52 ± 0.32
29	Octatriacontane	1.64 ± 0.24	2.04 ± 0.37	1.99 ± 0.29	1.33 ± 0.73	1.35 ± 0.37	0.98 ± 0.31	1.35 ± 0.28	1.15 ± 0.36	1.57 ± 0.21
30	17-methylheptatriacontane	2.90 ± 0.25	2.19 ± 0.21	2.99 ± 0.31	1.76 ± 1.08	1.89 ± 0.65	1.50 ± 0.37	2.56 ± 0.64	1.94 ± 0.57	1.95 ± 0.39
31	17, 21dimethylheptatriacontane	8.76 ± 0.91	1.90 ± 0.67	6.83 ± 0.78	3.45 ± 1.26	5.21 ± 1.71	4.97 ± 0.87	2.81 ± 1.46	4.01 ± 0.99	7.71 ± 1.13
32	13,17,21 trimethylheptatriacontane	1.46 ± 0.28	2.68 ± 1.21	3.40 ± 0.60	2.00 ± 0.89	2.48 ± 0.86	1.62 ± 0.37	2.00 ± 0.56	1.74 ± 0.53	2.32 ± 0.31
33	Tritetraconatne	2.24 ± 0.19	5.80 ± 3.38	3.23 ± 0.58	2.47 ± 0.89	2.48 ± 0.48	1.82 ± 0.53	2.24 ± 1.34	2.12 ± 1.57	4.84 ± 1.16
34	13-methyltritetracontane	0.70 ± 0.20	0.00	0.42 ± 0.16	0.07 ± 0.11	0.28 ± 0.19	0.17 ± 0.17	0.12 ± 0.09	0.46 ± 0.20	0.64 ± 0.26
35	unknown	0.78 ± 0.27	6.45 ± 0.61	1.09 ± 0.18	0.45 ± 0.33	0.46 ± 0.31	0.14 ± 0.13	0.62 ± 0.28	0.68 ± 0.29	0.69 ± 0.29
36	Tetratetracontane	0.92 ± 0.11	2.16 ± 0.35	0.92 ± 0.20	0.24 ± 0.23	0.49 ± 0.26	0.15 ± 0.15	0.97 ± 0.28	0.71 ± 0.30	0.54 ± 0.21
37	1-bromotetratetracontane	0.46 ± 0.31	0.00	0.65 ± 0.39	0.49 ± 0.36	0.85 ± 0.40	0.43 ± 0.24	0.98 ± 0.29	0.45 ± 0.35	1.23 ± 0.35
38	13-methyltetratetracontane	1.58 ± 0.19	0.00	1.40 ± 0.11	0.27 ± 0.25	0.46 ± 0.29	0.34 ± 0.21	0.88 ± 0.39	0.43 ± 0.20	1.33 ± 0.25
39	13, 17 dimethyltetratetracontane	1.30 ± 0.28	0.48 ± 0.48	1.17 ± 0.39	0.16 ± 0.20	0.63 ± 0.56	0.26 ± 0.28	0.07 ± 0.12	0.00	0.88 ± 0.82
40	unknown	1.12 ± 0.27	7.77 ± 2.58	1.61 ± 0.36	1.06 ± 0.59	1.21 ± 0.41	1.57 ± 0.86	0.95 ± 0.28	1.21 ± 0.50	1.18 ± 0.34

The 40 cuticular compounds identified and their percentage contribution (Mean ± SE), in the cuticular lipid extract sampled from field populations of Argentine ants for the 8 sites. Three nests were sampled per site (N = 18 runs per site). Elim (1+2) = Elim, Elim 3 = Elim 3, Bell = Bellville, Stell = Stellenbosch, Cal = Caledon, Port = Porterville, SW = Somerset West, Bredas = Bredasdorp, Jonk = Jonkershoek.

**Figure 5:**
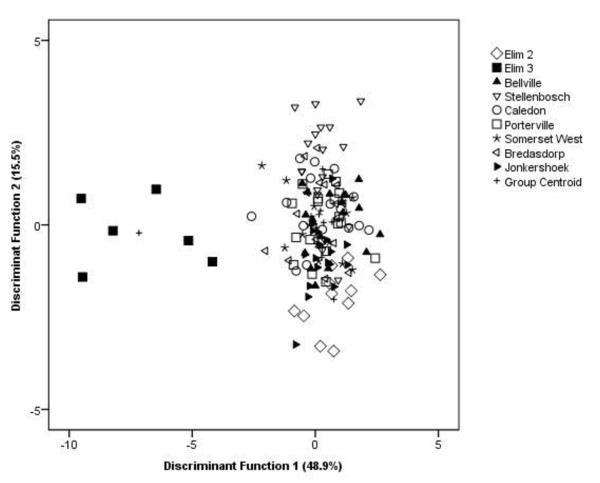
**Patterns of CHC profiles of Argentine ants from different sites**. Discriminant Analysis of Argentine ant cuticular hydrocarbons, based on nine principal component factors selected by Principal Component Analysis on forty CHC compounds extracted from field populations from eight sites in the Western Cape, South Africa. Ants from *Elim 3 *nest are significantly separated from all other ants in the remaining sites based on the CHC profiles.

The PCA produced nine principal components (PC) with eigenvalues larger than 1, explaining 74.5% of the total variance. A DA on these principal components significantly separated the Argentine ants into two groups based on the CHC profiles (Wilks' λ = 0.034 χ^2^= 46.86 d.f. = 72 *P *<0.0001, Figure 5). The ants from *Elim 3 *nest were the most aggressive, and they showed strong chemical divergence from all other ants used in this study (Figure 5). In the classification results of the DA, 57.4% of all CHC samples were correctly assigned to their respective groups and all *Elim 3 *ants were 100% correctly classified into their group. The ants from the different sites were separated along the Discriminant Function 1, and the compounds associated with this separation are the straight chained alkanes tetratriacontane and heptatriacontane, the methyl-branched alkanes 17-methylheptatriacontane and 13,17,21-trimethylheptatriacontane, as well as an unknown compound (peak 35, see Table 3).

## Discussion

This study is the first investigation of the population structure of Argentine ants in the Western Cape, South Africa using behavioural and chemical analyses. The chemical and behavioural data revealed that Argentine ants within the Western Cape are unicolonial and indicated the presence of at least two supercolonies that come into contact at Elim. Throughout this study ants were rarely aggressive to each other, except for all the interactions that included ants from *Elim 3 *where these ants were both behaviourally and chemically dissimilar from all other ants used in this study. These data are in keeping with the findings published on Argentine ant behaviour whereby ants from different supercolonies attack each other [20,22,30,33,42].

The CHC profiles of Argentine ants within the Western Cape consisted of the structural hydrocarbon classes found and identified in previous nestmate recognition studies of this ant [23,58]. We found that marked differences in CHC profiles, particularly for *Elim 3 *nest (Figure 5), resulted in maximum aggression, supporting the prediction that there is a negative relationship between chemical similarity and intraspecific aggression [55-58].

Argentine ant populations in the introduced ranges are characterised by widespread acceptance of non-nestmate conspecifics and genetic similarity among distant populations [20,23,30,31] as well as chemical resemblance across geographically separated locations [22,23]. Although this characteristic is attributed to reduced diversiy in genetic markers and nestmate recognition cues as a consequence of genetic bottleneck events experienced during introduction and establishment [20,30], the release from ecological constraints (i.e. pathogens, predators, parasites) and favourable environmental conditions in the introduced range have lead to the successful expansion of incipient colonies [6,16,30]. Consequently leading to the formation of the geographically vast supercolonies currently observed worldwide [65].

Argentine ant populations within the introduced range often have smaller supercolonies occurring within a larger supercolony, with nests from different supercolonies sometimes separated by distances less than 30m [66,67]. This pattern is similar for the South African population with nests from the two supercolonies interspersed within each other i.e. nests from different supercolonies occurring within short distances from each other. This type of distribution pattern was best explained by van Wilgenburg *et al.*, 2010. The initial establishment and spread of a large colony may prevent further establishment by propagules from different source populations, or if the propagules from other sources establish, their distribution will be largely limited by the population that established first [45]. This idea is supported by genetic data [68] for Elim which showed genetic structuring with "pockets" (of a divergent haplotype) surrounded by the larger supercolony (that included all other sites in this study), which is further supported our aggression data. The combination of these data from this study and the genetic data suggests that the observed aggression between ants from the two supercolonies is possibly an expression of underlying genetic differences. This is further supported by CHC congruency for the ants from the two populations. The pattern of chemical, behavioural and genetic differentiation between spatially close nests observed in this study are similar to that observed in Argentine ants from other parts of the introduced range [30,31,35,36,42]. The behavioural and chemical data in this study therefore offers support for at least two introductions of Argentine ants into South Africa.

Two recent studies showed the global distribution of Argentine ants is dominated by a single global dominant supercolony [41,45]. On continents and islands where this dominant supercolony exists, it is always the largest and most aggressive [45], displacing and outcompeting neighbouring supercolonies [20,30,32,69,70]. In their work, Van Wilgenburg *et al *(2010) included samples from Stellenbosch, Western Cape where they found that these ants do not form part of the global large supercolony and are likely to be an introduction from different source populations, as seen for other regions used in their study. Similarly, Vogel *et al.*, 2010 found that the Stellenbosch population was highly differentiated from the six supercolonies used in their study. Both these studies suggested that South African populations are likely a primary introduction from a different source population in the native range. However, two supercolonies have been identified in the Western Cape, and although Stellenbosch forms part of the large supercolony in South Africa, it is not known whether ants from the small supercolony may also originate from a primary introduction from the native range or from a secondary introduction from other introduced supercolonies not yet identified.

## Conclusions

Overall our results show that Argentine ants in the Western Cape, South Africa are unicolonial and form two supercolonies with a boundary at Elim. The observed behavioural differences between ants from the two supercolonies found in this study are possibly related to the phenotypic differences in the CHC profiles which are probably the expression of the underlying genetic differences. Our results are consistent with those found in earlier studies on the behaviour and colony structure of Argentine ants in other introduced ranges. These findings suggest that the similarities and differences in the phenotypic expression of CHCs and behaviour, among Argentine populations are important in the maintenance of unicoloniality and the formation of supercolonies.

## Authors' contributions

NPM conducted the sampling, experiments, analysis of data and drafted the manuscript. TCW helped develop the theoretical background for the study and the manuscript for publication. Both authors read and approved the final manuscript.
